# (*Z*)-*N*-[1-(Aziridin-1-yl)-2,2,2-tri­fluoro­ethyl­idene]-4-bromo­aniline

**DOI:** 10.1107/S1600536814007867

**Published:** 2014-04-12

**Authors:** Alexander S. Bunev, Maksim A. Vasiliev, Gennady I. Ostapenko, Alexander S. Peregudov, Victor N. Khrustalev

**Affiliations:** a Department of Chemistry and Chemical Technology, Togliatti State University, 14 Belorusskaya St, Togliatti 445667, Russian Federation; b NMR Laboratory, A.N. Nesmeyanov Institute of Organoelement Compounds, Russian Academy of Sciences, 28 Vavilov Street, B-334, Moscow 119991, Russian Federation; c X-Ray Structural Centre, A.N. Nesmeyanov Institute of Organoelement Compounds, Russian Academy of Sciences, 28 Vavilov Street, B-334, Moscow 119991, Russian Federation

## Abstract

The title compound, C_10_H_8_BrF_3_N_2_, crystallizes with two independent mol­ecules in the asymmetric unit, which can be considered as being related by a pseudo-inversion center, so their conformations are different; the corresponding N=C—N—C torsion angles are 54.6 (5) and −50.5 (5)°. In the crystal, mol­ecules related by translation in [001] inter­act through short inter­molecular Br⋯F contacts [3.276 (2) and 3.284 (2) Å], thus forming two types of crystallographically independent chains.

## Related literature   

For applications of aziridines, see: Tanner (1994 ▶); Remers & Iyengar (1995 ▶); Armstrong *et al.* (1996 ▶); Katoh *et al.* (1996 ▶); Schirmeister (1999*a* ▶,*b* ▶); McCoull & Davis (2000 ▶). For the crystal structures of related compounds, see: Chinnakali *et al.* (1998 ▶); McLaren & Sweeney (1999 ▶); Zhu *et al.* (2006 ▶); Moragas Solà *et al.* (2010 ▶).
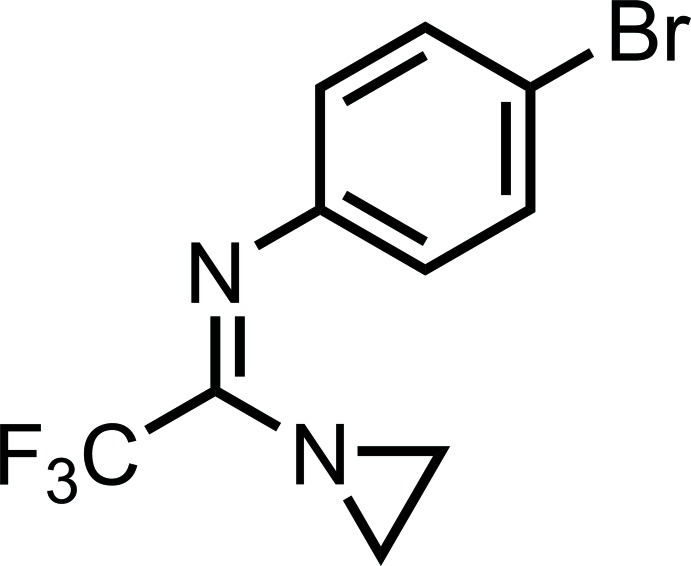


## Experimental   

### 

#### Crystal data  

C_10_H_8_BrF_3_N_2_
*M* *_r_* = 293.09
Monoclinic, 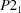

*a* = 11.642 (2) Å
*b* = 8.5455 (16) Å
*c* = 11.846 (2) Å
β = 116.106 (3)°
*V* = 1058.3 (3) Å^3^
*Z* = 4
Mo *K*α radiation
μ = 3.90 mm^−1^
*T* = 120 K
0.30 × 0.25 × 0.25 mm

#### Data collection  

Bruker APEXII CCD diffractometer
Absorption correction: multi-scan (*SADABS*; Bruker, 2003 ▶) *T*_min_ = 0.388, *T*_max_ = 0.442
13846 measured reflections
6146 independent reflections
5186 reflections with *I* > 2σ(*I*)
*R*_int_ = 0.043

#### Refinement  

*R*[*F* ^2^ > 2σ(*F* ^2^)] = 0.047
*wR*(*F* ^2^) = 0.118
*S* = 1.01
6146 reflections
289 parameters
1 restraint
H-atom parameters constrained
Δρ_max_ = 1.97 e Å^−3^
Δρ_min_ = −0.86 e Å^−3^
Absolute structure: Flack (1983 ▶), 2866 Friedel pairs
Absolute structure parameter: 0.025 (11)

### 

Data collection: *APEX2* (Bruker, 2005 ▶); cell refinement: *SAINT* (Bruker, 2001 ▶); data reduction: *SAINT* (Bruker, 2001 ▶); program(s) used to solve structure: *SHELXTL* (Sheldrick, 2008 ▶); program(s) used to refine structure: *SHELXTL* (Sheldrick, 2008 ▶); molecular graphics: *SHELXTL* (Sheldrick, 2008 ▶); software used to prepare material for publication: *SHELXTL* (Sheldrick, 2008 ▶).

## Supplementary Material

Crystal structure: contains datablock(s) global, I. DOI: 10.1107/S1600536814007867/cv5449sup1.cif

Structure factors: contains datablock(s) I. DOI: 10.1107/S1600536814007867/cv5449Isup2.hkl

Supporting information file. DOI: 10.1107/S1600536814007867/cv5449Isup3.cml

CCDC reference: 996162

Additional supporting information:  crystallographic information; 3D view; checkCIF report

## Figures and Tables

**Fig. 1.**
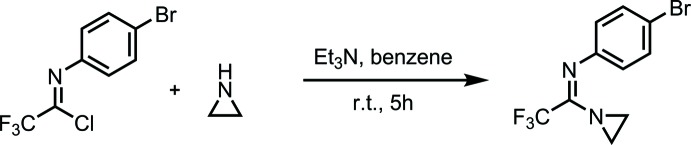
The synthesis of (Z)-N-[1-(aziridin-1-yl)-2,2,2-trifluoroethylidene]-4-bromoaniline.

**Fig. 2.**
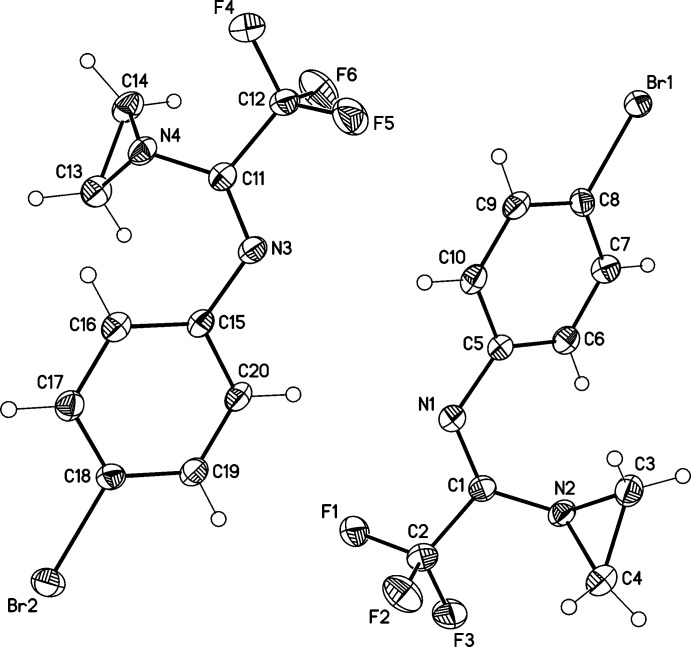
Molecular structure of I (two crystallographically independent molecules representing the different conformers are shown). Displacement ellipsoids are presented at the 50% probability level. H atoms are depicted as small spheres of arbitrary radius.

**Fig. 3.**
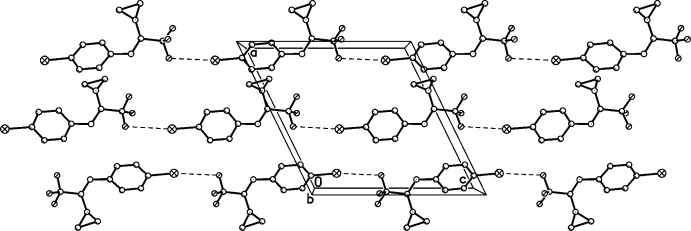
A portion of the crystal structure of I demonstrating the chains of the molecules extended along [001]. The intermolecular secondary Br···F interactions are depicted by dashed lines.

## supplementary crystallographic information

### 1. Comment

Aziridines are important heterocyclic compounds present in unusual natural products that display strong biological activity (Tanner, 1994; McCoull & Davis, 2000). For instance, azinomycines A and B isolated from the fermentation broth of *Streptomyces griseofuscus* and (+)-FR900482 isolated from the culture broth of *Streptomyces sandaensis* are potent antitumor antibiotics that exhibit exceptional activity against various types of mammalian solid tumors. Mitomycin C is an aziridine-containing antibiotic produced by Streptomyces caespitosus, whose antineoplastic activity is associated with the high reactivity of the strained aziridine ring (Remers & Iyengar, 1995; Armstrong *et al.*, 1996; Katoh *et al.*, 1996). Recently novel types of peptidic cysteine protease inhibitors containing aziridine-2,3-dicarboxylic acid have been designed and synthesized (Schirmeister, 1999*a*,*b*).

In this work, a new substituted aziridine, C_10_H_8_BrF_3_N_2_ (I), was prepared by the reaction *N*-(4-bromophenyl)-2,2,2-trifluoroethenecarbonimidoyl chloride with aziridine at room temperature (Figure 1), and its structure was unambiguously established by the X-ray diffraction study (Figure 2).

The title compound I crystallizes in chiral monoclinic space group *P*2_1_ with two crystallographically independent molecules in the unit cell. The two crystallographically independent molecules represent different conformers distinguishing by rotation of the aziridine substituent around the ordinary (CF_3_)C—N bond (the corresponding N1—C1—N2—C3 and N3—C11—N4—C13 totsion angles are 54.6 (5) and –50.5 (5)^o^, respectively). The bond lengths and angles in I are in a good agreement with those found in the related compounds (Chinnakali *et al.*, 1998; McLaren & Sweeney, 1999; Zhu *et al.*, 2006; Moragas Solà *et al.*, 2010). The molecule of I is the *Z*-isomer relative to the double C═N bond. The *p*-bromo-phenyl substituent is twisted by 38.42 (12) and 39.61 (10)% (for the two crystallographically independent molecules, respectively) relative to the double bond plane. Tha absolute structure of I was objectively determined by the refinement of Flack parameter, which has become equal to 0.025 (11).

In the crystal, the molecules of I form infinite chains along [001] by the intermolecular secondary Br1···F1^i^ (2.276 (2) Å) and Br2···F5^ii^ (3.284 (2) Å) interactions (Figure 3) [symmetry codes: (i) *x*, *y*, 1 + *z*; (ii) *x*, *y*, –1 + *z*]. The chains consist of the conformationally similar molecules and arranged at van der Waals distances.

### 2. Experimental

To a mixture of aziridine (1.29 mL, 1.075 g, 25 mmol), triethylamine (3.48 mL, 2.525 g, 25 mmol), and benzene (35 mL) was added slowly (20 min) a solution of *N*-(4-bromophenyl)-2,2,2-trifluoroethenecarbonimidoyl chloride in benzene (50 mL). The mixture was stirred for 5 h. The triethylamine hydrochloride was filtered, and the solvent was evaporated to give of crude product. Yield is 75%. The single-crystal of the product I was obtained by slow crystallization from EtOAc/Hexane (1:9). *M*.p. = 324–325 K. IR (KBr), ν/cm^-1^: 3408, 1715, 1629, 1492, 1295, 1200, 1155, 996, 828, 531. ^1^H NMR (600 MHz, DMSO-*d_6_*, 304 K): 7.56 (d, 2H, *J* = 8.2), 6.99 (d, 2H, *J* = 8.7), 2.21 (br. s, 4H). Anal. Calcd for C_10_H_8_BrF_3_N_2_: C, 33.54; H, 1.41. Found: C, 33.61; H, 1.52.

### 3. Refinement

All hydrogen atoms were placed in the calculated positions with C—H = 0.95 (aryl-H) and 0.99 (methylene-H) Å and refined in the riding model with fixed isotropic displacement parameters: *U*_iso_(H) = 1.2*U*_eq_(C)]. There are high positive residual densities of 1.44–1.97 eÅ^–3^ near the Br1 and Br2 centers (0.79–0.90 Å) due to considerable absorption effects which could not be completely corrected.

### Crystal data

**Table:**

C_10_H_8_BrF_3_N_2_	*F*(000) = 576
*M**_r_* = 293.09	*D*_x_ = 1.840 Mg m^−^^3^
Monoclinic, *P*2_1_	Mo *K*α radiation, λ = 0.71073 Å
Hall symbol: P 2yb	Cell parameters from 4691 reflections
*a* = 11.642 (2) Å	θ = 3.1–29.9°
*b* = 8.5455 (16) Å	µ = 3.90 mm^−^^1^
*c* = 11.846 (2) Å	*T* = 120 K
β = 116.106 (3)°	Prism, colourless
*V* = 1058.3 (3) Å^3^	0.30 × 0.25 × 0.25 mm
*Z* = 4	

### Data collection

**Table:**

Bruker APEXII CCD diffractometer	6146 independent reflections
Radiation source: fine-focus sealed tube	5186 reflections with *I* > 2σ(*I*)
Graphite monochromator	*R*_int_ = 0.043
φ and ω scans	θ_max_ = 30.0°, θ_min_ = 1.9°
Absorption correction: multi-scan (*SADABS*; Bruker, 2003)	*h* = −16→16
*T*_min_ = 0.388, *T*_max_ = 0.442	*k* = −12→11
13846 measured reflections	*l* = −16→16

### Refinement

**Table:**

Refinement on *F*^2^	Secondary atom site location: difference Fourier map
Least-squares matrix: full	Hydrogen site location: inferred from neighbouring sites
*R*[*F*^2^ > 2σ(*F*^2^)] = 0.047	H-atom parameters constrained
*wR*(*F*^2^) = 0.118	*w* = 1/[σ^2^(*F*_o_^2^) + (0.0665*P*)^2^] where *P* = (*F*_o_^2^ + 2*F*_c_^2^)/3
*S* = 1.01	(Δ/σ)_max_ = 0.001
6146 reflections	Δρ_max_ = 1.97 e Å^−^^3^
289 parameters	Δρ_min_ = −0.86 e Å^−^^3^
1 restraint	Absolute structure: Flack (1983), 2866 Friedel pairs
Primary atom site location: structure-invariant direct methods	Absolute structure parameter: 0.025 (11)

### Special details

**Table:**

Geometry. All esds (except the esd in the dihedral angle between two l.s. planes) are estimated using the full covariance matrix. The cell esds are taken into account individually in the estimation of esds in distances, angles and torsion angles; correlations between esds in cell parameters are only used when they are defined by crystal symmetry. An approximate (isotropic) treatment of cell esds is used for estimating esds involving l.s. planes.
Refinement. Refinement of F^2^ against ALL reflections. The weighted R-factor wR and goodness of fit S are based on F^2^, conventional R-factors R are based on F, with F set to zero for negative F^2^. The threshold expression of F^2^ > 2sigma(F^2^) is used only for calculating R-factors(gt) etc. and is not relevant to the choice of reflections for refinement. R-factors based on F^2^ are statistically about twice as large as those based on F, and R- factors based on ALL data will be even larger.

### Fractional atomic coordinates and isotropic or equivalent isotropic displacement parameters (Å^2^)

**Table:**

	*x*	*y*	*z*	*U*_iso_*/*U*_eq_	
Br1	0.11522 (3)	0.26312 (4)	1.18963 (3)	0.03080 (7)	
F1	0.09963 (18)	0.2247 (3)	0.45774 (18)	0.0409 (5)	
F2	−0.0174 (2)	0.4285 (3)	0.3927 (2)	0.0451 (5)	
F3	−0.1028 (2)	0.2017 (3)	0.35005 (19)	0.0444 (6)	
N1	0.0732 (2)	0.2970 (3)	0.6606 (2)	0.0273 (6)	
N2	−0.1541 (2)	0.2779 (3)	0.5405 (2)	0.0275 (5)	
C1	−0.0288 (3)	0.2907 (3)	0.5599 (3)	0.0251 (6)	
C2	−0.0130 (3)	0.2852 (4)	0.4393 (3)	0.0295 (7)	
C3	−0.2122 (3)	0.3864 (4)	0.5954 (3)	0.0319 (7)	
H3A	−0.1607	0.4771	0.6428	0.038*	
H3B	−0.2703	0.3426	0.6283	0.038*	
C4	−0.2560 (3)	0.3829 (4)	0.4556 (3)	0.0345 (8)	
H4A	−0.3408	0.3367	0.4024	0.041*	
H4B	−0.2311	0.4713	0.4170	0.041*	
C5	0.0744 (3)	0.2934 (4)	0.7808 (3)	0.0255 (6)	
C6	−0.0055 (3)	0.1941 (4)	0.8088 (3)	0.0283 (7)	
H6	−0.0683	0.1327	0.7443	0.034*	
C7	0.0070 (3)	0.1857 (4)	0.9306 (3)	0.0285 (7)	
H7	−0.0466	0.1183	0.9502	0.034*	
C8	0.0988 (2)	0.2770 (4)	1.0237 (3)	0.0267 (6)	
C9	0.1785 (3)	0.3738 (4)	0.9972 (3)	0.0274 (6)	
H9	0.2406	0.4358	1.0619	0.033*	
C10	0.1679 (3)	0.3806 (4)	0.8765 (3)	0.0279 (7)	
H10	0.2245	0.4448	0.8587	0.034*	
Br2	0.42565 (3)	0.56843 (4)	0.34093 (3)	0.03331 (7)	
F4	0.6460 (2)	0.5903 (4)	1.18097 (19)	0.0506 (6)	
F5	0.44343 (19)	0.6038 (3)	1.07301 (19)	0.0455 (6)	
F6	0.5344 (2)	0.3820 (3)	1.1287 (2)	0.0497 (6)	
N3	0.4603 (2)	0.5260 (3)	0.8663 (2)	0.0261 (6)	
N4	0.6881 (2)	0.5003 (3)	0.9894 (2)	0.0273 (6)	
C11	0.5624 (3)	0.5129 (4)	0.9676 (3)	0.0241 (6)	
C12	0.5469 (3)	0.5233 (4)	1.0885 (3)	0.0293 (7)	
C13	0.7334 (3)	0.3884 (4)	0.9255 (3)	0.0320 (7)	
H13A	0.7994	0.4238	0.8999	0.038*	
H13B	0.6710	0.3124	0.8677	0.038*	
C14	0.7710 (3)	0.3724 (4)	1.0628 (3)	0.0342 (8)	
H14A	0.7318	0.2866	1.0901	0.041*	
H14B	0.8603	0.3982	1.1222	0.041*	
C15	0.4617 (3)	0.5305 (3)	0.7482 (3)	0.0242 (6)	
C16	0.5513 (3)	0.6135 (4)	0.7248 (3)	0.0284 (7)	
H16	0.6205	0.6634	0.7923	0.034*	
C17	0.5408 (3)	0.6246 (4)	0.6031 (3)	0.0283 (7)	
H17	0.6023	0.6813	0.5872	0.034*	
C18	0.4399 (3)	0.5519 (4)	0.5065 (3)	0.0263 (6)	
C19	0.3487 (3)	0.4687 (4)	0.5270 (3)	0.0294 (7)	
H19	0.2802	0.4184	0.4592	0.035*	
C20	0.3586 (3)	0.4601 (4)	0.6479 (3)	0.0280 (7)	
H20	0.2952	0.4061	0.6627	0.034*	

### Atomic displacement parameters (Å^2^)

**Table:**

	*U*^11^	*U*^22^	*U*^33^	*U*^12^	*U*^13^	*U*^23^
Br1	0.02611 (10)	0.03998 (15)	0.02471 (11)	0.00583 (12)	0.00971 (9)	0.00366 (12)
F1	0.0358 (8)	0.0569 (14)	0.0337 (8)	0.0102 (8)	0.0188 (7)	0.0010 (8)
F2	0.0660 (11)	0.0346 (11)	0.0429 (9)	0.0019 (9)	0.0315 (9)	0.0065 (8)
F3	0.0423 (10)	0.0577 (13)	0.0293 (8)	−0.0130 (10)	0.0122 (8)	−0.0137 (9)
N1	0.0243 (9)	0.0302 (13)	0.0260 (10)	−0.0010 (9)	0.0099 (8)	−0.0010 (9)
N2	0.0209 (9)	0.0305 (12)	0.0267 (10)	−0.0031 (10)	0.0066 (8)	−0.0002 (10)
C1	0.0245 (10)	0.0251 (14)	0.0239 (11)	−0.0043 (10)	0.0088 (9)	−0.0046 (10)
C2	0.0301 (12)	0.0265 (15)	0.0278 (12)	−0.0035 (11)	0.0090 (10)	−0.0042 (11)
C3	0.0260 (12)	0.0347 (16)	0.0347 (14)	0.0017 (12)	0.0130 (11)	−0.0019 (12)
C4	0.0251 (12)	0.0389 (17)	0.0343 (14)	0.0007 (13)	0.0082 (11)	0.0002 (13)
C5	0.0211 (10)	0.0302 (15)	0.0234 (11)	0.0010 (10)	0.0082 (9)	0.0009 (10)
C6	0.0250 (12)	0.0275 (14)	0.0305 (13)	−0.0021 (11)	0.0104 (10)	−0.0007 (11)
C7	0.0291 (12)	0.0238 (14)	0.0307 (13)	−0.0041 (11)	0.0112 (11)	0.0021 (11)
C8	0.0255 (10)	0.0297 (14)	0.0276 (11)	0.0030 (12)	0.0143 (9)	0.0019 (11)
C9	0.0192 (11)	0.0283 (14)	0.0300 (13)	−0.0010 (10)	0.0064 (10)	−0.0046 (11)
C10	0.0201 (10)	0.0293 (14)	0.0321 (13)	−0.0019 (11)	0.0093 (10)	0.0018 (12)
Br2	0.03588 (13)	0.03795 (15)	0.02514 (11)	0.00254 (13)	0.01254 (10)	0.00202 (12)
F4	0.0362 (9)	0.0816 (18)	0.0296 (8)	−0.0126 (11)	0.0106 (7)	−0.0142 (11)
F5	0.0434 (9)	0.0601 (15)	0.0381 (9)	0.0166 (10)	0.0227 (8)	0.0032 (9)
F6	0.0759 (12)	0.0382 (11)	0.0497 (10)	0.0016 (11)	0.0411 (9)	0.0083 (9)
N3	0.0213 (9)	0.0281 (13)	0.0247 (10)	−0.0008 (9)	0.0064 (8)	0.0010 (9)
N4	0.0192 (9)	0.0300 (12)	0.0285 (11)	0.0033 (9)	0.0067 (9)	0.0002 (10)
C11	0.0231 (11)	0.0207 (12)	0.0267 (12)	−0.0004 (10)	0.0093 (10)	−0.0006 (10)
C12	0.0237 (12)	0.0372 (16)	0.0232 (12)	0.0025 (11)	0.0068 (10)	−0.0014 (11)
C13	0.0301 (12)	0.0297 (15)	0.0361 (14)	0.0028 (12)	0.0144 (11)	−0.0051 (12)
C14	0.0228 (12)	0.0367 (16)	0.0347 (15)	0.0057 (12)	0.0049 (11)	0.0074 (13)
C15	0.0184 (10)	0.0257 (14)	0.0260 (11)	0.0019 (9)	0.0073 (9)	0.0011 (10)
C16	0.0234 (11)	0.0303 (15)	0.0285 (12)	−0.0025 (10)	0.0088 (10)	−0.0019 (11)
C17	0.0232 (11)	0.0294 (14)	0.0301 (13)	0.0018 (11)	0.0095 (10)	0.0076 (11)
C18	0.0234 (11)	0.0309 (15)	0.0221 (11)	0.0038 (11)	0.0077 (9)	0.0029 (11)
C19	0.0215 (11)	0.0363 (16)	0.0274 (12)	−0.0006 (11)	0.0080 (10)	−0.0029 (12)
C20	0.0186 (10)	0.0313 (14)	0.0302 (13)	−0.0033 (11)	0.0073 (10)	−0.0033 (12)

### Geometric parameters (Å, º)

**Table:**

Br1—C8	1.893 (3)	Br2—C18	1.899 (3)
F1—C2	1.335 (4)	F4—C12	1.322 (4)
F2—C2	1.335 (4)	F5—C12	1.328 (4)
F3—C2	1.322 (4)	F6—C12	1.329 (4)
N1—C1	1.261 (3)	N3—C11	1.269 (4)
N1—C5	1.418 (4)	N3—C15	1.407 (4)
N2—C1	1.377 (4)	N4—C11	1.373 (4)
N2—C3	1.459 (4)	N4—C13	1.456 (4)
N2—C4	1.474 (4)	N4—C14	1.464 (4)
C1—C2	1.520 (4)	C11—C12	1.522 (5)
C3—C4	1.504 (5)	C13—C14	1.493 (5)
C3—H3A	0.9900	C13—H13A	0.9900
C3—H3B	0.9900	C13—H13B	0.9900
C4—H4A	0.9900	C14—H14A	0.9900
C4—H4B	0.9900	C14—H14B	0.9900
C5—C10	1.392 (4)	C15—C16	1.387 (4)
C5—C6	1.403 (5)	C15—C20	1.399 (4)
C6—C7	1.387 (5)	C16—C17	1.396 (5)
C6—H6	0.9500	C16—H16	0.9500
C7—C8	1.389 (4)	C17—C18	1.375 (4)
C7—H7	0.9500	C17—H17	0.9500
C8—C9	1.378 (4)	C18—C19	1.386 (5)
C9—C10	1.382 (4)	C19—C20	1.387 (5)
C9—H9	0.9500	C19—H19	0.9500
C10—H10	0.9500	C20—H20	0.9500
			
C1—N1—C5	122.5 (3)	C11—N3—C15	121.8 (3)
C1—N2—C3	122.7 (3)	C11—N4—C13	123.6 (3)
C1—N2—C4	122.7 (3)	C11—N4—C14	122.7 (3)
C3—N2—C4	61.7 (2)	C13—N4—C14	61.5 (2)
N1—C1—N2	130.5 (3)	N3—C11—N4	131.5 (3)
N1—C1—C2	115.9 (3)	N3—C11—C12	115.8 (3)
N2—C1—C2	113.4 (2)	N4—C11—C12	112.6 (2)
F3—C2—F2	107.0 (2)	F4—C12—F5	107.3 (3)
F3—C2—F1	107.2 (3)	F4—C12—F6	106.8 (3)
F2—C2—F1	106.2 (3)	F5—C12—F6	106.5 (3)
F3—C2—C1	112.8 (3)	F4—C12—C11	112.6 (3)
F2—C2—C1	111.2 (3)	F5—C12—C11	112.1 (2)
F1—C2—C1	112.0 (2)	F6—C12—C11	111.2 (3)
N2—C3—C4	59.7 (2)	N4—C13—C14	59.5 (2)
N2—C3—H3A	117.8	N4—C13—H13A	117.8
C4—C3—H3A	117.8	C14—C13—H13A	117.8
N2—C3—H3B	117.8	N4—C13—H13B	117.8
C4—C3—H3B	117.8	C14—C13—H13B	117.8
H3A—C3—H3B	114.9	H13A—C13—H13B	115.0
N2—C4—C3	58.6 (2)	N4—C14—C13	59.0 (2)
N2—C4—H4A	117.9	N4—C14—H14A	117.9
C3—C4—H4A	117.9	C13—C14—H14A	117.9
N2—C4—H4B	117.9	N4—C14—H14B	117.9
C3—C4—H4B	117.9	C13—C14—H14B	117.9
H4A—C4—H4B	115.1	H14A—C14—H14B	115.0
C10—C5—C6	119.6 (3)	C16—C15—C20	119.3 (3)
C10—C5—N1	117.7 (3)	C16—C15—N3	123.4 (3)
C6—C5—N1	122.4 (3)	C20—C15—N3	116.9 (3)
C7—C6—C5	120.0 (3)	C15—C16—C17	120.6 (3)
C7—C6—H6	120.0	C15—C16—H16	119.7
C5—C6—H6	120.0	C17—C16—H16	119.7
C6—C7—C8	119.3 (3)	C18—C17—C16	118.9 (3)
C6—C7—H7	120.4	C18—C17—H17	120.5
C8—C7—H7	120.4	C16—C17—H17	120.5
C9—C8—C7	121.1 (3)	C17—C18—C19	121.7 (3)
C9—C8—Br1	120.2 (2)	C17—C18—Br2	118.7 (2)
C7—C8—Br1	118.7 (2)	C19—C18—Br2	119.6 (2)
C8—C9—C10	119.9 (3)	C18—C19—C20	119.1 (3)
C8—C9—H9	120.1	C18—C19—H19	120.5
C10—C9—H9	120.1	C20—C19—H19	120.5
C9—C10—C5	120.2 (3)	C19—C20—C15	120.3 (3)
C9—C10—H10	119.9	C19—C20—H20	119.9
C5—C10—H10	119.9	C15—C20—H20	119.9
			
C5—N1—C1—N2	−0.8 (5)	C15—N3—C11—N4	−0.2 (5)
C5—N1—C1—C2	−176.2 (3)	C15—N3—C11—C12	175.1 (3)
C3—N2—C1—N1	54.6 (5)	C13—N4—C11—N3	−50.5 (5)
C4—N2—C1—N1	129.6 (4)	C14—N4—C11—N3	−125.8 (4)
C3—N2—C1—C2	−130.0 (3)	C13—N4—C11—C12	134.1 (3)
C4—N2—C1—C2	−54.9 (4)	C14—N4—C11—C12	58.8 (4)
N1—C1—C2—F3	146.9 (3)	N3—C11—C12—F4	−146.2 (3)
N2—C1—C2—F3	−29.3 (4)	N4—C11—C12—F4	30.0 (4)
N1—C1—C2—F2	−92.9 (3)	N3—C11—C12—F5	−25.1 (4)
N2—C1—C2—F2	91.0 (3)	N4—C11—C12—F5	151.1 (3)
N1—C1—C2—F1	25.8 (4)	N3—C11—C12—F6	94.0 (3)
N2—C1—C2—F1	−150.3 (3)	N4—C11—C12—F6	−89.9 (3)
C1—N2—C3—C4	112.6 (3)	C11—N4—C13—C14	−112.2 (3)
C1—N2—C4—C3	−112.5 (3)	C11—N4—C14—C13	113.6 (3)
C1—N1—C5—C10	−145.7 (3)	C11—N3—C15—C16	−41.6 (4)
C1—N1—C5—C6	40.9 (4)	C11—N3—C15—C20	145.2 (3)
C10—C5—C6—C7	1.3 (5)	C20—C15—C16—C17	−1.3 (5)
N1—C5—C6—C7	174.6 (3)	N3—C15—C16—C17	−174.3 (3)
C5—C6—C7—C8	0.3 (5)	C15—C16—C17—C18	0.1 (5)
C6—C7—C8—C9	−0.9 (5)	C16—C17—C18—C19	0.2 (5)
C6—C7—C8—Br1	−179.6 (2)	C16—C17—C18—Br2	179.6 (2)
C7—C8—C9—C10	−0.2 (5)	C17—C18—C19—C20	0.7 (5)
Br1—C8—C9—C10	178.5 (2)	Br2—C18—C19—C20	−178.7 (2)
C8—C9—C10—C5	1.9 (5)	C18—C19—C20—C15	−1.9 (5)
C6—C5—C10—C9	−2.5 (5)	C16—C15—C20—C19	2.2 (5)
N1—C5—C10—C9	−176.0 (3)	N3—C15—C20—C19	175.7 (3)
